# Trends in Postpartum Hemorrhage Prevalence and Comorbidity Burden: Insights from the ENACT Network Aggregated Electronic Health Record Data

**DOI:** 10.21203/rs.3.rs-5041092/v1

**Published:** 2024-09-26

**Authors:** Malarkodi J. Samayamuthu, Olga Kravchenko, Wei-Hsuan Lo-Ciganic, Eugene M. Sadhu, Seonkyeong Yang, Shyam Visweswaran, Vanathi Gopalakrishnan

**Affiliations:** University of Pittsburgh; University of Pittsburgh; University of Pittsburgh; University of Pittsburgh; University of Pittsburgh; University of Pittsburgh; University of Pittsburgh

## Abstract

The goal of this study was to assess trends in postpartum hemorrhage (PPH), its risk factors, and maternal comorbidity burden in the United States using aggregate data from the Evolve to Next-Gen Accrual to Clinical Trials (ENACT) network. This federated network employs interactive querying of electronic health record data repositories in academic medical centers nationwide. We conducted repeated annual cross-sectional analyses to evaluate PPH occurrence and comorbidities across various ethnoracial and sociodemographic groups, starting with a large cohort of 1,287,675 unique delivery hospitalizations collected from 22 ENACT sites between 2005 and 2022. During this time, there was a statistically significant increasing trend in the prevalence of PPH, rising from 5,634 to 10,504 PPH per 100,000 deliveries (P*_trend_* <0.001). Our findings revealed a continuous upward trend in PPH rates that remained consistent among women with ≥ 1 comorbid conditions (P*_trend_* <0.001) and those with ≥ 1 maternal risk factor (P*_trend_* <0.001). This result aligns with prior studies and extends beyond the time periods previously reported. Overall, Native Hawaiian or Other Pacific Islander women had the highest PPH prevalence (~ 13%), followed by Asian (9.8%), American Indian or Alaska Native (8.9%), multirace (8.6%), Black or African American (8.4%) and White (7.4%) women. The top PPH risk factor identified was placenta previa or accreta, while the top comorbidity was antepartum hemorrhage / placental abruption. The most common cause of PPH, namely uterine atony, was prevalent in ENACT data. Our analysis highlights significant ethnoracial disparities and underscores the need for targeted preventative interventions.

## Introduction

Health sciences research is increasingly leveraging real-world data from various sources to generate evidence on patient health status and healthcare delivery. Electronic health records (EHRs), which record patient contacts, treatments, and clinical activities within healthcare systems, are a valuable source of real-world data and are increasingly used in epidemiological, clinical, and translational studies. Two primary approaches have emerged for assembling EHR (Electronic Health Record) data across multiple institutions: centralized and federated. In the centralized approach, EHR data from participating institutions are transferred to a central data repository, where the data are standardized and quality controlled. This centralized storage facilitates sophisticated analytics with patient-level data. However, there can be significant delays in data availability for analysis and potential scalability issues as data volume grows. Examples of research programs that use this approach include the All of Us Research Program^1^ and the National COVID Cohort Collaborative (N3C) initiative.^2^ In contrast to the centralized approach, the federated approach keeps each participating institution’s data locally while harmonizing the data across institutions to a common standard. Federated networks can often provide access to a larger volume of data with shorter time lags and pose lower security risks to healthcare systems. However, accessing and aggregating data from multiple institutions can be time-consuming or require sophisticated technology to ensure consistency and accuracy. Programs that use a federated approach include the National Patient-Centered Clinical Research Network (PCORnet),^3^ the Consortium for Clinical Characterization of COVID-19 by EHR (4CE),^4^ and the Evolve to Next-Gen Accrual of patients to Clinical Trials (ENACT) network.^5^ The ENACT network is unique among these programs in that it employs an interactive querying approach for local data repositories that does not require programming knowledge.

The ENACT network consists of local EHR data repositories that use either the Informatics for Integrating Biology at the Bedside (i2b2) or the Observational Medical Outcomes Partnership (OMOP) data models, which are linked via the Shared Health Research Information Network (SHRINE) platform.^6^ The network is a collaboration of more than 50 data-contributing institutions (ENACT sites) affiliated with academic medical research centers.^5–7^ The SHRINE platform facilitates federated queries across the network that are constructed using medical ontologies in key domains, including demographics, diagnoses, procedures, medications, laboratory test results, and visit characteristics. To ensure that data is updated, each site updates its repository at least once a month. While the SHRINE platform enables querying of all sites on the network, not all may respond to every query due to factors such as data repository downtime, network traffic, and other technical issues. Furthermore, the set of responding sites may vary with each query, especially when executing a sequence of multiple queries. While the ENACT network has the potential to accelerate real-world evidence generation from large-scale analysis of EHRs, a first step is to evaluate the alignment of evidence generated from the network via the SHRINE platform with findings from prior research that used individual-level data.

We conducted this evaluation herein via a case study on postpartum hemorrhage (PPH), a critical condition that significantly impacts maternal morbidity and mortality worldwide.^8–13^ Our primary goal was to evaluate the consistency between the evidence derived from SHRINE-generated aggregated data and that previously published using individual-level data. The American College of Obstetricians and Gynecologists (ACOG) defines PPH as either cumulative blood loss exceeding 1000 mL or blood loss accompanied by signs or symptoms of hypovolemia within 24 hours of childbirth.^14^ Although the incidence of PPH in the United States (US) increased from 2.7% in 2000 to 4.3% in 2019, maternal mortality due to PPH has decreased since the 1980s, with a reduction to 1.7 deaths per 100,000 live births in 2009.^14^ Despite this decline, in 2019, PPH accounted for 13.7% of pregnancy-related deaths in the US.^15^

The etiology of PPH has been categorized by the “Four Ts”: Tone (uterine atony), Trauma (laceration, rupture), Tissue-related (retained products of conception), and Thrombin-induced (coagulopathies), with uterine atony identified as the most common cause, accounting for approximately 80% of cases.^14^ Key maternal risk factors for PPH include advanced maternal age, obesity, multiple gestation, previous PPH history, uterine surgery, complications during pregnancy such as preeclampsia, labor induction, prolonged labor, macrosomia, and pre-existing conditions (e.g., coagulation disorders).^8,14,16^ Emergency cesarean sections and other intra-partum factors also increase the likelihood of PPH.^16^ Identifying these risk factors is crucial for targeted preventative measures and improving maternal health outcomes.^17^ We have used the SHRINE platform to obtain comprehensive statistics on PPH, including the proportion of deliveries affected by PPH, associated comorbidities and risk factors. A repeated annual cross-sectional analysis was conducted to examine trends in PPH incidence and the burden of comorbidity among affected women. We hypothesized that insights from the SHRINE platform would be consistent with findings from prior research using data from the Agency for Healthcare Research and Quality’s (AHRQ’s) National (Nationwide) Inpatient Sample (NIS).^8^ Our study is vital for validating SHRINE’s effectiveness as a resource for generating real-world evidence in health sciences, particularly in maternal health research and conditions as critical as PPH.

## Methods

### Data Sources, Quality Assessment and Site Selection

We used SHRINE to query the ENACT network to obtain patient counts relevant for our analyses. The EHR data in the network is de-identified to ensure patient privacy and comply with HIPAA (Health Insurance Portability and Accountability) regulations, and SHRINE returns aggregate counts of unique patients meeting query criteria. To ensure privacy and estimate accuracy, these counts are rounded to the nearest five and further obfuscated by adding or subtracting up to 10 counts.^18^ Due to obfuscation, the same query may return slightly different counts when rerun, though these variations are typically negligible especially when the counts are high. The University of Pittsburgh’s Institutional Review Board (IRB) has approved the usage of the ENACT network for a wide range of research under protocol number STUDY19080059.

**Figure 1:**
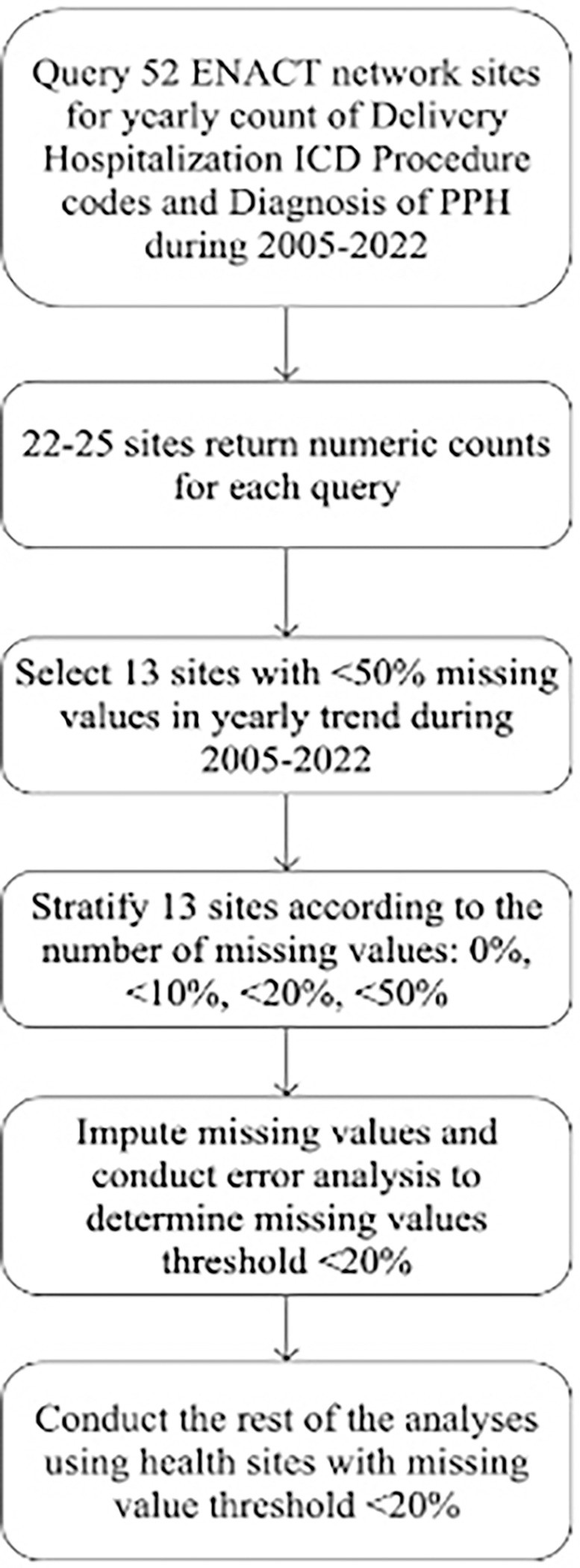
Workflow and exclusion criteria for inclusion of sites.

The queries were submitted to all sites on the ENACT network, resulting in responses from 22 to 25 sites for some or all submitted queries. To qualify for inclusion in the analyses, sites were required to meet specific inclusion criteria, ensuring data quality and completeness. Sites were required to have no more than 50% missing annual data points, allowing for up to nine years without data within the eighteen-year span from 2005 to 2022 for each query. Additionally, sites were excluded if more than 50% of annual data points showed values significantly smaller than adjacent years by at least two orders of magnitude. This indicated potential data errors or absence of relevant EHRs, meaning the data did not accurately reflect the actual count requested by the query. Finally, thirteen sites met the criteria and were included in the subsequent analyses. Data from the year 2023 were excluded from our analyses due to incomplete data contributions from the included sites at the time of extraction. All further analyses were conducted using data exclusively from the thirteen identified sites to ensure consistency. Figure 1 illustrates the workflow used to assess data quality and identify suitable sites.

### Study Design and Data Extraction

**Figure 2:**
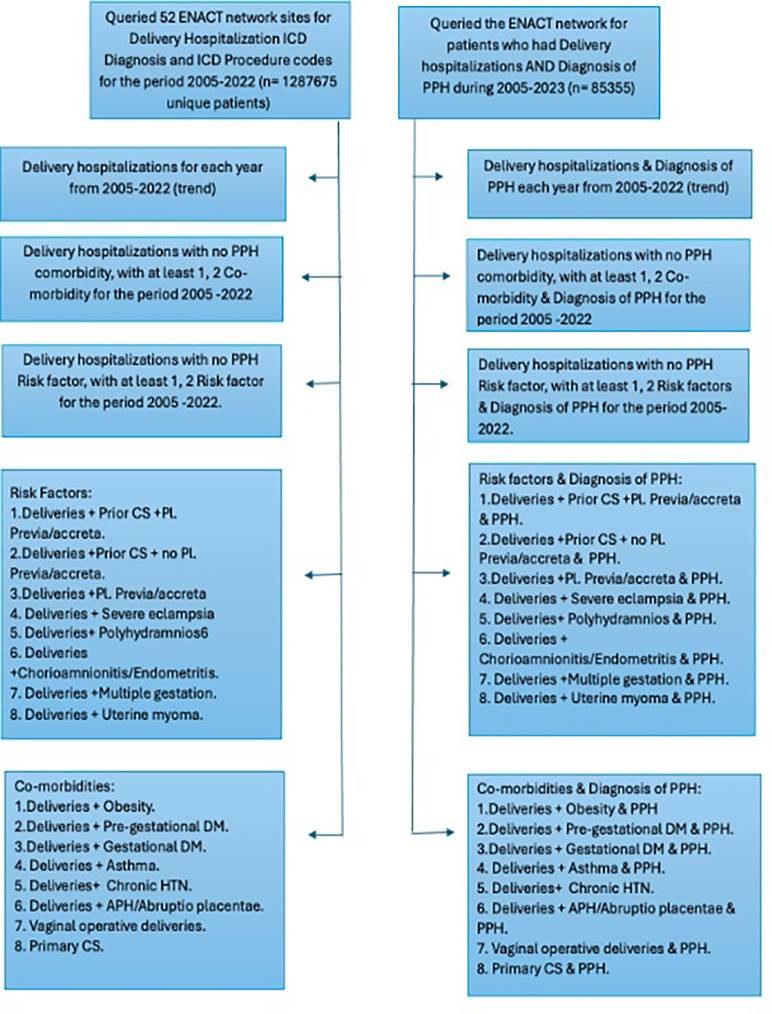
Query sequence for obtaining counts from the ENACT network containing 52 sites at the time of this study. ICD - International Classification of Diseases, PPH -Postpartum Hemorrhage, CS - Cesarean Section, PL. Previa - Placenta Previa, DM - Diabetes Mellitus, HTN - Hypertension. APH - Antepartum hemorrhage. The n on the left hand-side and right hand-side is reported from 22 and 20 sites, respectively. Using the above query sequence, we were able to obtain initial data from 22 sites, and process them further as shown in the Figure 1 workflow. Supplementary spreadsheet file attached to this manuscript contains ICD codes used in the queries constructed for accurate data extraction.

We conducted repeated annual cross-sectional analyses using SHRINE-generated aggregate counts from EHR data spanning from 2005 to 2022. First, we identified unique individuals who had delivery hospitalizations in each calendar year using a pre-existing algorithm.^19^ Then, within this cohort, we identified deliveries with PPH using the International Classification of Diseases, Ninth and Tenth Revisions, Clinical Modification (ICD-9-CM / ICD-10-CM) codes (see Supplemental spreadsheet file for a list of codes used to identify PPH). Women with delivery hospitalizations without PPH served as a comparator group. Figure 2 depicts the sequence of queries used in SHRINE used to create the dataset for the analyses of PPH among delivery hospitalizations. There are two query sequences: one for all delivery hospitalizations and another for delivery hospitalizations with a PPH diagnosis.

### Outcome Measures

Our primary outcome of interest was the annual incidence rate of PPH among delivery hospitalizations, with a focus on examining trends in incidence rates over the entire study period. Acknowledging the potential limitation of overestimation due to use of aggregated data, for each year, we compared demographics and clinical characteristics between the two groups: deliveries with PPH and deliveries without PPH. The demographics included women 15–54 years old stratified by 10-year age intervals, and race (White, Black or African American, Asian, American Indian or Alaska Native, Native Hawaiian or Other Pacific Islander, multi-race, and unknown). It is important to note that the maternal age provided in the dataset is the age at the time of data extraction (i.e., current age), not the age at the time of delivery. Due to constraints in constructing queries in SHRINE, it is not possible to obtain age at a specific moment of time in the past. Therefore, maternal age could not be considered a variable in our analysis.

As for clinical characteristics, we assessed a number of comorbid conditions known to potentially complicate both pregnancy and delivery and maternal risk factors for PPH. The comorbid conditions included abruptio placentae, asthma, chronic hypertension, diabetes, gestational diabetes mellitus, obesity, pre-gestational diabetes mellitus, primary cesarean section (CS), and vaginal operative deliveries. Additionally, maternal risk factors included prior CS, placenta previa, placenta accreta spectrum disorder, uterine rupture, severe eclampsia, endometritis, multiple gestation, and uterine leiomyoma. The PPH interventions included a range of medical and surgical approaches, such as the administration of medications used in PPH management, blood transfusion and peripartum hysterectomy. Furthermore, we categorized PPH according to the “Four Ts” causes (i.e., tone, trauma, tissue, thrombin) as secondary outcomes. We distinguished between two types of traumatic causes: putative causes such as obstetric trauma or lacerations that might lead to PPH and confirmed causes where PPH was clearly caused by trauma. The PPH outcomes, comorbidities and risk factors were reported as counts and proportions.

### Data Analyses

Our initial analysis focused on assessing the extent of missing data in the extracted dataset and determining an appropriate threshold for missing values. First, we calculated the PPH incidence rates for sites with no missing values (0% missing data) to establish a baseline trend. The annual incidence rate was calculated as follows: $Incidence\;Rate=\frac{\mathrm{Number}\;\mathrm{of}\;\mathrm{PPH}\;\mathrm{cases}}{\mathrm{Number}\;\mathrm{of}\;\mathrm{Deliveries}}\times 100,000$. Contributing sites were then grouped based on the percentage of missing data into four categories: 0%, < 10%, < 20% and < 50% of missing data. For each group, missing values were imputed using linear regression when there were two or more consecutive missing years or for a boundary year. For single-year missing data, the counts of the preceding and subsequent years were averaged. We then calculated the annual incidence rates for each group and compared these rates with the baseline data using visual analysis and the Pearson correlation coefficients between pairs of normalized curves. The correlation between 0% and < 10%, as well as 0% and < 20% datasets were found to be strong, with r=0.941 and r=0.923, respectively, while correlation between 0% and < 50% was found to be moderate with r=0.765. Based on this analysis we chose < 20% missing values threshold to maximize data inclusion without compromising data quality. This reduced the number of sites used in all further analyses to 8.

The trends in PPH incidence rates were examined within subgroups defined by race and presence of comorbidities and maternal risk factors. To assess the significance of the trend in PPH prevalence over time, we employed the non-parametric Mann-Kendall trend test.^20^ The Mann-Kendall test was chosen for its robustness against data irregularities and its ability to analyze trends without assuming a specific data distribution. Further, we assessed the prevalence of PPH in each co-morbidity and risk factors subgroups.

We computed the prevalence of comorbidities for individuals who had PPH deliveries (comorbidity | PPH), where the comorbidities included obesity, pre-gestational diabetes mellitus, gestational diabetes mellitus, asthma, chronic hypertension, antepartum hemorrhage / abruptio placentae, vaginal operative deliveries, and primary CS. We also computed the reciprocal prevalence of PPH delivery given these comorbidities (PPH | comorbidity). Data analyses were conducted using the Excel software, version 4.3.2 (2023-10-31) for spreadsheet management and Python 3.7.3 and Jupyter Notebook 6.5.4.

## Results

The initial dataset comprised 1,287,675 unique delivery hospitalizations collected from 22 ENACT sites between 2005 and 2022. After data quality assessment, the analyses included data from 13 sites, totaling 886,725 unique deliveries. Table 1 shows demographic characteristics and risk factors for PPH of total deliveries and deliveries involving PPH for individuals from the 13 sites. Most deliveries were among White women (498,560), with 37,015 out of 57,970 (63.85%) of them involving PPH. Black or African American women had 97,445 total deliveries, with 8,175 out of 57,970 (14.1%) involving PPH. Maternal race data was contributed by 12 sites, due to missing data from one site. It is also important to note that the breakdown by age is only given for rough estimation of the percentages of PPH occurring within age groups. We purposefully do not use age breakdown data in our analyses as mentioned in the Methods section. The data obtained for risk factors, comorbidities, and race/ethnicities yielded robust trends that are reported herein. It is interesting to note in the Table 1 within the maternal race groupings, if we observe the percentages of PPH deliveries, all except for the White subgroup are showing slight increases when compared to the percentage of non-PPH deliveries for their subgroup. It is heartening to see a decreasing percentage of PPH deliveries for the White subgroup when compared to the percentage of non-PPH deliveries in these data, though much more research and its translation into clinical care will be needed to serve women of all these various subgroups. Precision in managing PPH risk is therefore a clear and significant need.

**Figure 3:**
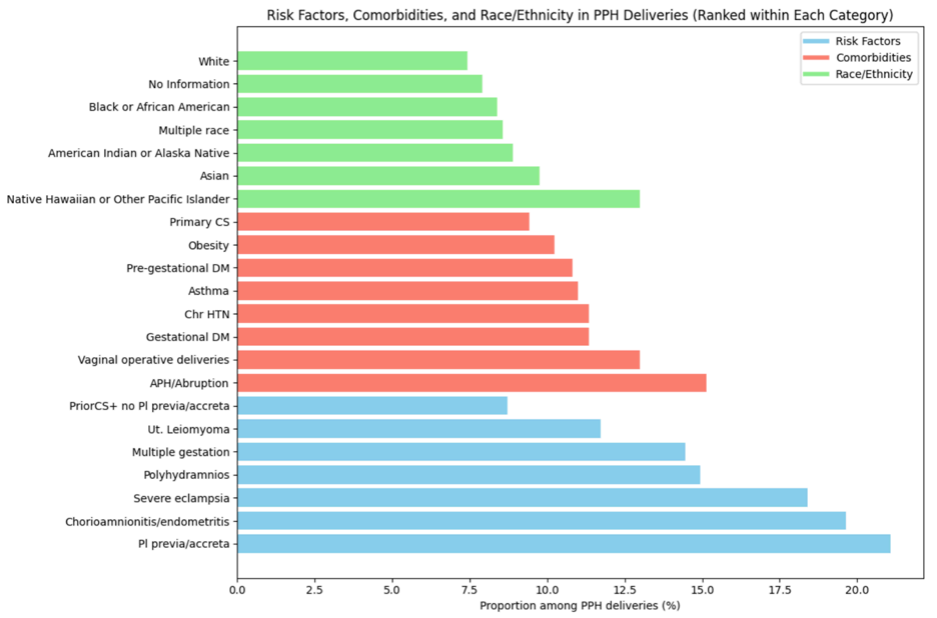
Ranked percentage of various risk factors (blue), comorbidities (red), and race/ethnicity (green) associated with PPH deliveries during the period of 2005–2022, across 8 sites.

The overall prevalence of PPH among total deliveries was 11.22% during the period from 2005 to 2022. As shown in Fig. 3, among ethnoracial groups, PPH prevalence was highest among Native Hawaiian or Other Pacific Islander women (12.98%), followed by Asian women (9.75%), American Indian or Alaska Native women (8.9%), multirace (8.6%), Black or African American women (8.39%) and White women (7.42%). Women with prior CS without placenta previa / placenta accreta had a PPH prevalence of 8.70%, while those with placenta previa / placenta accreta had the highest prevalence of 21.09%. Women with severe eclampsia had a prevalence of 18.40%, women with chorioamnionitis / endometritis had a prevalence of 9.63%, and women with multiple gestations had a prevalence of 14.44%. In terms of comorbidities, the highest prevalence of PPH occurred in women with antepartum hemorrhage (APH) / abruption (15.13%), and women with vaginal operative deliveries (12.98%).

**Figure 4:**
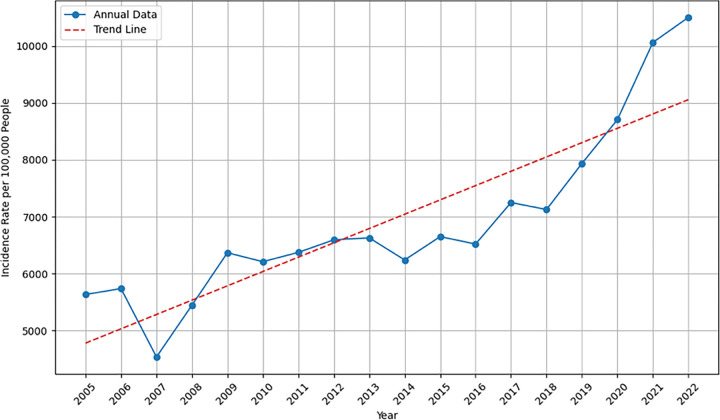
Incidence of postpartum hemorrhage (PPH) per100,000 deliveries from 2005 to 2022 for sites with no more than 20% missing values (blue) and corresponding trend line Ptrend<0.001 (dashed red; P_trend_ <0.001), across 8 sites.

Figure 4 shows PPH incidence rate trend for the years 2005 to 2022. During this time, there was a statistically significant increasing trend in the prevalence of PPH, rising from 5,634 to 10,504 PPH per 100,000 deliveries (P*_trend_* <0.001). This trend remained consistent among individuals with ≥ 1 comorbid conditions (P*_trend_* <0.001) and those with ≥ 1 maternal risk factors (P*_trend_* <0.001).

**Figure 5:**
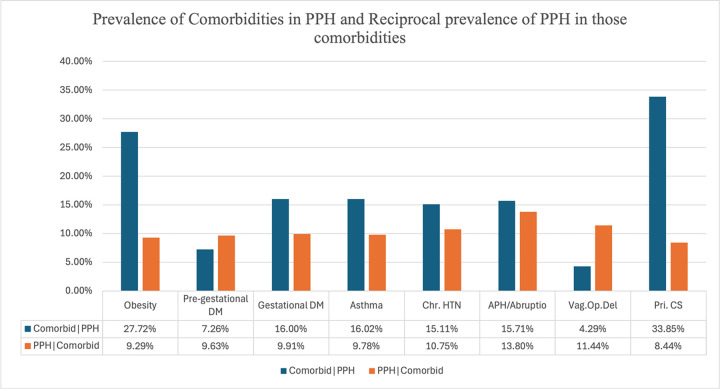
Prevalence of comorbidities in postpartum hemorrhage (PPH) deliveries and reciprocal prevalence of PPH in comorbid conditions, across 8 sites. DM - Diabetes mellitus, Chr. HTN - Chronic hypertension, APH / Abruptio - Antepartum hemorrhage/Abruptio placentae, Vag. Op. Del - Vaginal operative delivery, Pri. CS -Primary Cesarean section.

The sites contributing to the analysis in Fig. 4 have between 0 to 20% of missing values (no more than 4 years of missing values per site). To choose the missing values threshold, error analysis was conducted, that compared curves with different number of missing values. We observed that increasing the number of imputed missing values does not significantly affect the trend line and the data’s overall trends are preserved (data not shown). We chose 20% as the missing value threshold to balance the number of missing values with the number of contributing sites taken into consideration, preserving data integrity. Figure 5 illustrates the prevalence of various comorbidities in PPH deliveries and the reciprocal prevalence of PPH within those comorbid conditions. Conditions such as obesity, gestational diabetes, asthma, chronic hypertension, and APH / abruptio placentae have a high prevalence in PPH deliveries. The reciprocal prevalence of PPH is particularly high among women with APH / abruptio placentae (13.80%), vaginal operative deliveries (11.44%), and chronic HTN (10.75%). While primary CS has the highest prevalence of PPH among comorbid conditions (33.85%), the prevalence of PPH in women undergoing primary CS is relatively lower at 8.44%. Conversely, obesity, with a high prevalence in PPH deliveries (27.72%), also shows a significant reciprocal PPH prevalence (9.29%).

**Figure 6:**
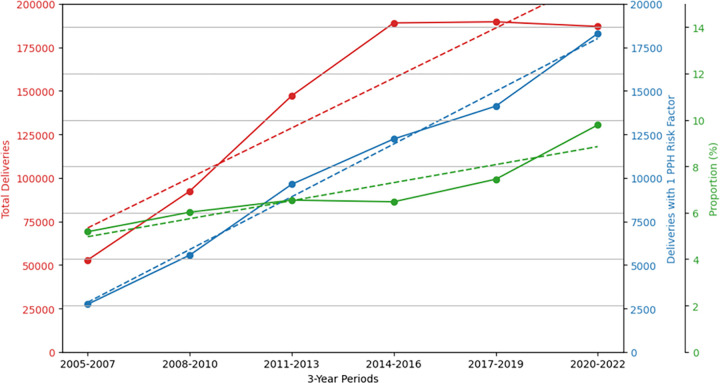
Total number of deliveries (red solid line) and count of deliveries with 1 PPH risk factor (blue dashed line) for years 2005 to 2022, extracted in 3-year periods. Total deliveries (red solid line) with trend (red dashed line, P_trend_ = 0.056), deliveries with 1 PPH risk factor (blue solid line) with trend (blue dashed line, P_trend_ = 0.003), and proportion of deliveries with 1 PPH risk factor (green solid line) with trend (green dashed line, P_trend_ = 0.017) from 2005 to 2022 in 3-year periods, across 8 sites.

Additionally, we examined the trend in deliveries with 1 PPH risk factor present. Figure 6 illustrates the trends in total deliveries, deliveries with 1 PPH risk factor, and proportion of deliveries with 1 PPH risk factor as a percentage of the total deliveries. From 2005 to 2022, there is a noticeable increase in both the total number of deliveries (P = 0.056) and deliveries with 1 PPH risk factor (P = 0.003). At the same time, the proportion of deliveries with 1 PPH risk factor shows an upward trend (5.2% in 2005 to 9.8% in 2022, P_trend_=0.017), indicating a rising prevalence of PPH risk factors relative to the total number of deliveries.

Table 2 presents the causes and interventions of PPH in the years 2005 to 2022 observed in our dataset. Among the causes, the prevalence of atonic PPH was identified in 53,325 deliveries, while traumatic PPH was significantly higher, with 363,260 deliveries. Note, that while there are multiple causes associated with obstetric trauma / perineal laceration, not all instances lead to PPH. For example, only 13,515 of the women with trauma subsequently developed PPH. This distinction is captured as follows: “traumatic cause” refers to obstetric trauma or lacerations that may lead to PPH; and “trauma leading to PPH” refers PPH that was definitely caused by trauma. Tissue-related causes such as retained placenta were observed in 21,945 deliveries, and thrombin – induced PPH in 1,835 deliveries. In terms of outcomes and interventions, PPH resulted in 1,280 hysterectomies. Additionally, PPH led to 8,920 women undergoing surgery. There were 11,000 deliveries where PPH required manipulative procedures, and 6,000 deliveries necessitated blood transfusions. The most common intervention was the administration of medications for PPH, which occurred in 37,720 deliveries.

## Discussion

The main objective of this study was to assess the trends in PPH and maternal comorbidity burden in the US using the aggregated data from the ENACT network. Our analysis revealed a rising trend in PPH, consistent with the trend observed in a previously published study covering the 2009–2019 period. This study relied on the NIS data, which comprises a 20% sample of all delivery hospitalizations in the US and is collected annually by the AHRQ’s Healthcare Cost and Utilization Project.^8^ By including data from before 2009 and after 2019, our study corroborates previously observed trends and shows that the upward trajectory of PPH continues beyond the earlier study period. Given that PPH is life-threatening, our findings underscore the urgent need to identify individuals at risk of PPH for timely preventative interventions.

Additionally, this analysis validates the utility of the data in the ENACT network for conducting research efficiently. Despite the data being available only in aggregate counts, we established reproducible data quality procedures and analysis workflows that confirm the reliability of the data in the network for similar analyses. Our querying methods were refined several times after discussions with the ENACT technical team to ensure data accuracy. This validation is significant, demonstrating that the data in the network, when processed through rigorous data quality procedures, can serve as a valuable resource for future research.

Based on the incidence rates of PPH across the various races and sociodemographic factors in our results, we found that the Native Hawaiian or Other Pacific Islander subgroup had the highest incidence rate of PPH (~ 13%), followed by Asian women (~ 10%), American Indian or Alaska Native (~ 9%), and Black or African American (~ 8%). Our findings are consistent with prior studies that confirmed similar disparities using more rigorous statistical methods applied to individual-level EHR and chart review data.^21–23^

Our analyses identified the top risk factor as placenta previa / placenta accreta and the top comorbidity as APH / abruptio placentae. Compared to the results from Corbetta-Rastelli et al.,^8^ our results show that uterine atony, the most common cause of PPH, is also evident in our results. Furthermore, we examined the causes and interventions for PPH that were not addressed in the Corbetta-Rastelli et al. study.

In sum, our analysis yields actionable findings regarding ethnoracial disparities, showing that Native Hawaiian or Other Pacific Islander, Asian, American Indian or Alaska Native, and Black or African American women have increasing prevalence of PPH. The rank ordering of comorbidities and risk factors also emphasizes the importance of identifying and managing multiple gestation, prior CS, gestational diabetes, and chronic hypertension. These comorbidities and risk factors can help to inform future studies in designing personalized risk prediction models for PPH.

## Limitations

Our study has several noteworthy limitations. First, to preserve patient privacy, the patient counts provided in SHRINE are obfuscated. This obfuscation may introduce some level of inaccuracy in the data, potentially affecting the precision of the analysis results. Because the overall counts for each query are generally very large (in the tens or hundreds of thousands or millions), any small count should be suspected of being generated solely by automatic obfuscation and should be thoroughly examined for validity.

The technology used in ENACT calculates the number of unique patients meeting query criteria, which may lead to undercounting if multiple occurrences of the same diagnostic code are present. For instance, the technology might not account for second, third, or subsequent delivery instances for the same woman, which may affect the accuracy of the trends and outcomes related to multiple deliveries. Depending on the research question, this can be mitigated by choosing an appropriate timeframe for querying the condition of interest.

In this study, we have utilized only ICD-9 and ICD-10 codes for procedures, based on methodologies established in previous studies. However, some ENACT sites may not have mapped their delivery procedures to ICD (International Classification of Diseases) codes. All sites have mapped procedures to CPT^®^ (Current Procedural Terminology) codes, which were not included in the phenotypes used for this study. The exclusion of CPT^®^ codes may lead to incomplete data capture, potentially missing some delivery procedures.

Finally, we cannot study individuals longitudinally and we lack detailed information of temporal relationship and confounding factors to infer causality. Despite this, the data obtained in SHRINE is a rich source of timely real-world evidence to support subsequent individual-level data analysis.

## Conclusions

This case study illustrates the use of aggregated EHR data from a federated network of sites across the US to assess trends in PPH among various racial, ethnic, and age groups. We found a clearly increasing trend in PPH incidence for the period 2005–2022. Our finding underscores the need for better approaches to more accurately identify women at an elevated risk for PPH, while accounting for diverse ethnoracial groups. Given that prior Cesarean deliveries can increase risk of PPH in subsequent deliveries, women and their obstetricians will need to carefully assess and ensure medical necessity of Cesarean deliveries to avoid future risk of PPH.^24^ Also, ethnic minorities may need better screening through carefully designed studies to ensure that appropriate preventative measures are prescribed during pregnancy, while also preparing adequately for managing blood loss should PPH occur. Carefully planned logistical interventions such as preparing for blood type and expert obstetricians’ availability, are important for managing women at elevated risk for PPH. As sufficient data is collected for local management of PPH across the globe, results from studies such as this will enable better risk management to prevent and manage this significant event in women’s lives, which can impact their mental and physical health. In the future, the limitations of aggregated data will be overcome by conducting studies on cohorts of patients with individual-level data (e.g., the global maternal and newborn health eCohorts^25^) that will yield more precise data to verify the risk factors and co-morbidities identified herein.

## Figures and Tables

**Table 1:** Demographics of total deliveries, postpartum hemorrhage (PPH) deliveries, comorbidities, and risk factors across 13 sites.

	Total Deliveries (n = 886725)	PPH Deliveries (n = 69745)	PPH Deliveries (%)	Non-PPH Deliveries (%)
Age (13 sites) *				
Median Age Group	35–44	45–54		
**Age Group**				
<18	59915	< 20	N/A	N/A
>=18	821550	69690	100	100
18–54	800380	68930	98.60	97.34
55+	20975	980	1.40	2.66
Maternal race**	**Total Deliveries (n = 743335)**	**PPH Deliveries** **(n = 57970)**		
American Indian or Alaska Native	3710	330	0.57	0.50
Asian	53315	5200	8.97	7.17
Black or African American	97445	8175	14.10	13.11
Multiple race	4320	370	0.64	0.58
Native Hawaiian or Other Pacific Islander	1695	220	0.38	0.23
No Information	84290	6660	11.49	11.34
White	498560	37015	63.85	67.07
**No. of PPH risk factors**				
0 PPH Risk factor	556840	29795	29.48	52.44
deliveries + 1 PPH Risk factor	324855	41225	40.78	28.22
deliveries + 2 PPH Risk factor	224430	30055	29.74	19.34
**No. of PPH co-morbidities**				
deliveries + 0 PPH co-morbidity	323350	21810	17.08	30.38
deliveries + 1 PPH co-morbidity	441920	52745	41.30	39.21
deliveries + 2 PPH co-morbidity	354785	53140	41.62	30.41
**PPH risk factors**	**deliveries with at least 1 risk factor**			
Deliveries + Prior CS + no Pl previa/accreta	124835	10865	18.79	33.49
Deliveries + Pl previa/accreta	61550	12980	22.44	14.27
Deliveries + Severe eclampsia	31965	5880	10.17	7.66
Deliveries + Polyhydramnios	27740	4145	7.17	6.93
Deliveries + Chorioamnionitis/endometritis	62005	12170	21.04	14.64
Deliveries + Multiple gestation	45485	6570	11.36	11.43
Deliveries + Ut. Leiomyoma	44575	5230	9.04	11.56
**Comorbidities in PPH**	**deliveries with at least 1 comorbidity**			
Deliveries + Obesity	198980	20370	19.93	21.22
Deliveries + Pre-gestational DM	51230	5545	5.42	5.43
Deliveries + Gestational DM	100265	11385	11.14	10.56
Deliveries + Asthma	108455	11920	11.66	11.47
Deliveries + Chr HTN	97145	11020	10.78	10.23
Deliveries + APH/Abruptio	82220	12440	12.17	8.29
Vaginal operative deliveries	28465	3410	3.34	2.98
Primary CS	277055	26135	25.56	29.80


* Age represents the age of women at the time of data extraction. This variable has been shown only for rough estimates. All variations in the numbers for age breakdowns are due to obfuscation methods to preserve patient privacy.

** Maternal race data were contributed by 12 sites, due to missing race data in one of the contributing sites.

N/A indicates that the data negligible and likely can be attributed solely to obfuscation.

**Table 2:** Causes and interventions of postpartum hemorrhage (PPH) for years 2005 to 2022. Traumatic causes: obstetric trauma or lacerations that may cause PPH. Trauma leading to PPH: definite cases of PPH which occurred after trauma.

Causes	Count
Prevalence of atonic PPH	53325
Traumatic causes PPH	363260
Trauma leading to PPH	13515
Tissue PPH	21945
Thrombin PPH	1835
**Outcomes/Interventions**	
PPH and hysterectomy	1280
PPH and all surgeries	8920
PPH and procedures	11000
PPH and blood transfusion	6000
PPH and associated medication use	37720
